# Case Report: Congenital Arthrogryposis and Unilateral Absences of Distal Arm in Congenital Zika Syndrome

**DOI:** 10.3389/fmed.2021.499016

**Published:** 2021-04-13

**Authors:** Silvina Noemí Contreras-Capetillo, José Rafael Palma-Baquedano, Nina Valadéz-González, Pablo Manrique-Saide, Hirian Alonso Moshe Barrera-Pérez, Doris Pinto-Escalante, Norma Pavía-Ruz

**Affiliations:** ^1^Hospital General Agustín O'Horán, Secretaría de Salud de Yucatán, Yucatan, Mexico; ^2^Centro de Investigaciones Regionales Dr. Hideyo Noguchi, Universidad Autónoma de Yucatán, Yucatan, Mexico; ^3^Facultad de Medicina, Universidad Anáhuac Mayab, Yucatán, Mexico; ^4^Campus de Ciencias Biológicas y Agropecuarias, Universidad Autónoma de Yucatán, Yucatan, Mexico; ^5^Servicios particulares de Anatomopatología, ANAPAT, Yucatan, Mexico

**Keywords:** Zika virus, birth defects, congenital infection, arthrogryposis, microcephaly, sequence, disruption

## Abstract

Zika virus was recognized as a teratogen in 2015, when prenatal Zika infection was associated with neonatal microcephaly. The transmission, virulence, tropism, and consequences of Zika virus infection during pregnancy are currently studied. Decreased neural progenitor cells, arrest in neuronal migration and/or disruption of the maturation process of the fetus central nervous system have been associated. Congenital Zika Syndrome produces a fetal brain disruption sequence resulting in structural brain abnormalities, microcephaly, intracranial calcifications, fetal akinesia and arthrogryposis. Vascular abnormalities like unique umbilical artery and decreased cerebral vascular flow have been described in some patients. This article reports a Zika positive patient with sequence of fetal brain disruption, arthrogryposis and absence of distal third of the right forearm. This report expands the clinical observations of congenital Zika syndrome that may be related to disruptive vascular events.

## Introduction

Since the identification of the Zika virus (ZIKV) in a rhesus monkey in 1947 and its isolation in humans in 1954, this virus has caused outbreaks in different populations from 2007 to 2013, and recently in 2015 in Brazil, with different public health impacts (1–4). ZIKV infection in humans is related to blood dyscrasias such as thrombocytopenia, Guillain Barre Syndrome and structural morphological abnormalities in fetus of infected pregnant mothers (5–8). But, up to 80% of those infected will course asymptomatic. ZIKV is transmitted by the bite of infected *Aedes aegypti* mosquito, sexual or vertical transmission during pregnancy, through blood transfusions, among others (3, 9–12). ZIKV is a flavivirus with two identified, Asian and African, lineages. Its RNA genome (10.8 kb) encodes for a 3,419-amino acid polyprotein which forms a capsid (C), a membrane precursor (prM), a wrap (E), and 7 non-structural proteins (NS1, NS2A, NS2B, NS3, NS4A, NS4B y NS5) (13). Revers transcription polymerase chain reaction (RT-PCR) and reverse transcription quantitative polymerase chain reaction (RT-qPCR) are reliable tests for detection of viral ZIKV RNA in serum or urine of infected patients but they are limited because of fast decline of virus presence in these tissues (1 to 2 weeks post-infection). Tests for ZIKV-specific IgM antibodies in serum are also used as a diagnostic evidence for ZIKV infection expanding the diagnostic opportunity for several months, but cross-reactivity with other flaviviruses like dengue, must be taken into account, especially in endemic areas (14, 15).

The pathogenicity of ZIKV is related to cellular events like apoptosis, vascular damage, restriction in the cell maturity, and the signal cascade activation, but its virulence and cellular pathology is not totally elucidated (16–18). ZIKV interferes with the neural development through decreased neural progenitor cells, arrest in neuronal migration and/or disruption of the maturation process of the fetus central nervous system (CNS). This is clinically translated into microcephaly, lissencephaly, and others brain abnormalities (19–22). The objective of this article is to report one patient with brain sequence disruption, arthrogryposis and absence of the distal segment of the right arm with ZIKV RNA detected in the cerebrospinal fluid.

## Case Report

During the 2016 ZIKV outbreak in Merida, México, a 27-year-old woman in the third trimester of pregnancy was referred to medical geneticist because multiple malformations detected in the fetus. Informed consent was obtained for sampling, clinical evaluations, and for the publication report. Exploring the medical records, she reported unquantified fever, preauricular nodes, pruritus and rash in the shoulder girdle and thorax in the first trimester when the pregnancy was unnoticed. No serological tests were performed for ZIKV at that time. Ultrasound was performed at 16.4 weeks of gestation with report of fetal growth within normal ranges; but at 23 weeks of gestation, the fetal hands were not identified. At 27.4 weeks of gestation, fetus was reported with microcephaly (DBP 58 mm); nuchal thickening, ventriculomegaly, hemisphere hypoplasia and cerebellar vermis were detected in the brain, and micrognathia, right radial aplasia, and arthrogryposis were also reported at that time.

**Figure 1:**
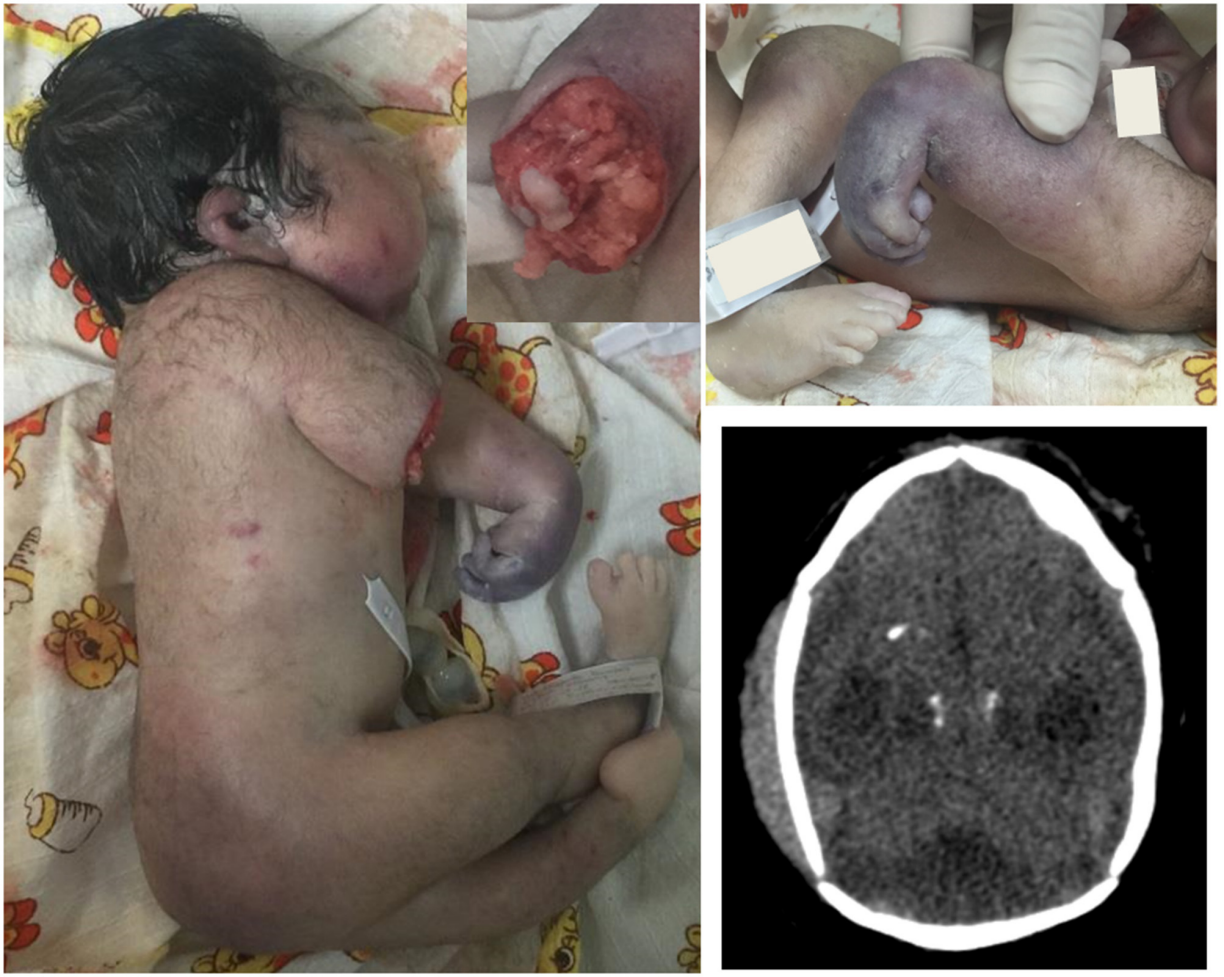
**(A)** In this picture it is appreciate the patient phenotype with arthrogryposis and the absence of the distal part of the right arm The frontal view of the arm injury is inserted in the upper part of the photo. **(B)** Observe cyanotic coloration in the distal left arm and hand in comparison of the foot. **(C)** Axial-cut cranial tomography showing subcortical calcifications.

A stillbirth with generalized subcutaneous edema was obtained via cesarean section at 35 weeks of gestation. At physical exploration showed craniofacial disproportion, microcephaly, irregular anterior and lower posterior hairline. Posterior sloping of the forehead and hypertelorism were observed. The nasal bridge, the nostrils and the filtrum were normal. Retrognathia and normal oral cavity were found. The ears were cupped with low implantation and thickened helix. The shoulders were short, with internal rotation and presented limitation to abduction. The left upper limb presented an extended elbow with limitation to the reduction, pronation arm, flexed wrist, non-reducible hand with cyanotic coloration. The upper right limb was conformed only to the proximal third of the arm. At this level, soft tissue defect was found with the presence of an irregular cutaneous line, exposure of subcutaneous tissue and the humeral condyle, no tissue bleeding was detected (Figure 1). The lower extremities presented limitation to hip abduction, knee extension and flexion of both feet. The genitalia anatomy showed 1 cm penis and a complete rough scrotum without testes inside.

**Figure 2:**
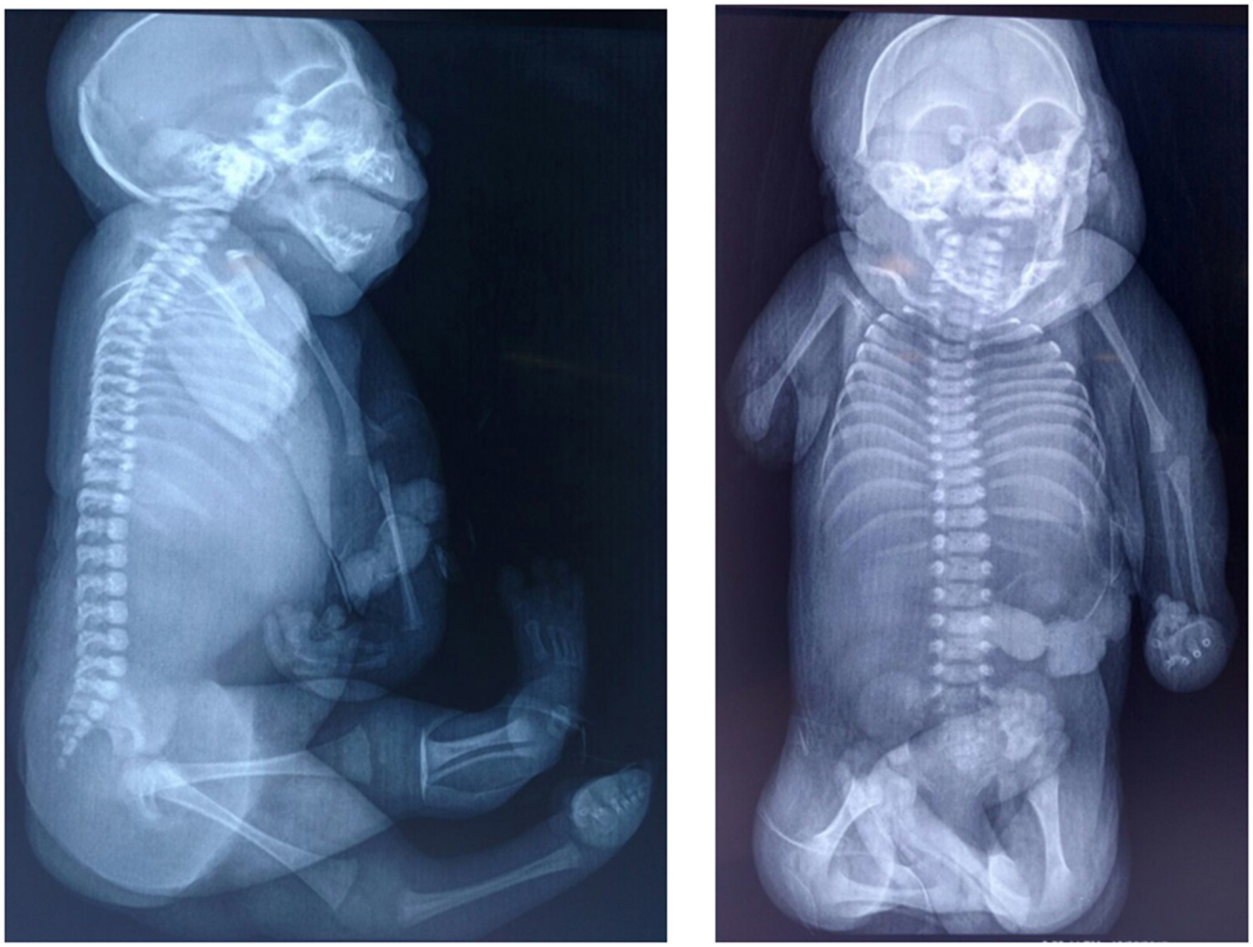
X-ray imaging. In both image it appreciate skull with everted sutures and partial collapse of the cranial bones. Asymmetry is observed in the length of the right and left humerus.

On the skull x-ray, everted sutures and partial collapse of the cranial bones with a hypoplastic occipital was observed. The radiograph of the upper extremities shows a right humerus shorter than the left, with preserved of the distal region of the humerus (Figure 2). Computational axial tomography reported subcortical calcifications, lissencephaly, ventriculomegaly, and generalized cortical degeneration. The karyotype was normal, 46, XY. The serological test for toxoplasma, rubella, cytomegalovirus and herpes virus were negative in the mother and the patient. The RT-PCR for ZIKV/Dengue/Chikungunya in the patient's cerebrospinal fluid detected the presence of Zika viral RNA (23). Autopsy was not authorized.

## Discussion

The Zika virus outbreak in Brazil in 2015 became emergent due to catastrophic consequences in infected newborns during the prenatal period (7, 8). Current research investigates the virulence and pathogenicity of the African and Asian ZIKV lineages to understand why this teratogenic effect was not observed in earlier outbreaks (24, 25). Epidemiology during the outbreak in Brazil allowed to observe that: (1) Pregnant women infected with Zika were asymptomatic or symptomatic as well as the general population; (6) (2) Not all pregnant women with ZIKV infection had perinatal complications or their products had congenital abnormalities, it was estimated that up to 5–10% of these women had children with morphological abnormalities (8), (3) Structural abnormalities found in fetus and newborns were related to brain tissue disruption sequence and growth restriction (7, 21, 22). (4) Establishing a conclusive diagnosis of Congenital Zika Syndrome (CZS) is a challenge due to the prolonged time between acute (symptomatic or asymptomatic) maternal infection and the time when fetal abnormalities are detected (8), and finally, (5) Although the presence of viral RNA has been demonstrated for prolonged periods in serum, urine, semen and other tissues of infected patients, to stablish ZIKV diagnosis is still a challenged because is related to optimal RNA recovery methods. These methods are under investigations actually (14, 15).

In CZS, a fetal brain disruption sequence (FBDS) was described, thus numerous events would produce variable findings in brain imaging tests. The sequence of disruption is a congenital, static morphological abnormality, caused by the developmental failure of a body structure that had the normal (genetic) developmental potential. The embryological or fetal moment at which the tissue is interrupted or the development determines subsequent destruction; may therefore, be heterogeneous (26). Until now, different etiologies of FBDS are described, being the infections and vascular injuries more frequent (22). ZIKV interferes with neural development through the decrease of neural progenitor cells, the arrest in neuronal migration and/or disruption of the CNS maturation process (19, 20, 27). The involvement of neuronal stem cells in human fetuses through non-structural proteins (NS4B and NS4A) has been associated with the inhibition of Akt-mTor signaling that participates in brain development (28). Depending on the damage to brain tissue, microcephaly can be observed and subsequent fetal skull collapse would result (22). Microcephaly, intracranial calcifications and brain disruption were the most frequent abnormalities shown in SCZ (21).

As a consequence of brain or peripheral nerves damage, can occur decrease in fetal movements and joint contractures (arthrogryposis) in consequence. It is known while limitation of fetal movement is earlier in gestational age and lower range of joint movement happens, the greater joint involvement and contractures will be observed at birth (29). This was one of the proposed mechanisms of the most severely affected patients with CZS (30). Arthrogryposis can occur as an isolated manifestation or as part of other genetic syndromes. Its etiology is not well-known; however, abnormalities in connective, nervous, muscular and vascular tissue have been related as possible pathological mechanisms (29).

In the patient here described, the contractures observed in the left arm were internal shoulder rotation, extended elbow and flexed wrist corresponding to amyoplasia, which is the most frequent arthrogryposis (31). However, the left arm was cyanotic from the middle part of the forearm to the acroterminal region and the wrist was hyperflexed with overlapping fingers with no reducible position. The isquemic pattern in the left hand could suggest vascular disruption. No constrictor rings were detected (32). The transverse terminal deficiency of the right limb is suspected of being lost during the last 8 weeks of pregnancy, because an obstetric ultrasound reported radial aplasia at 27 weeks gestation. In the distal stump, the skin was irregular and no bleeding was observed. Also, granulation tissue was found in the stump and fetal remains were not founded inside the uterus so that, this lost limb was not considered a traumatic event. The right arm injury presented in this patient was found different from those reported for gangrene, ischemia and necrosis, as well as in the cases described of compartmental neonatal syndrome. Various reviews of sequences of amniotic bands show constriction rings, with hypoplasia of the post-ring region; even so, post-ring lesion usually has normal skin (31, 33). Teratogens associated with disruptive events in limbs have been described for a long time. Even so, limb amputations in uterus are infrequently reported (29–34). In ZIKV infection, cell cycle arrest and apoptosis happen in neuronal cell, but abnormalities in the density and vascular diameter of the brain had been reported (21). In addition, patients with abnormalities of cerebral flow and umbilical cord with single artery also were reported, so the vascular damage in others ZIKV affected tissues should still be clarified.

## Data Availability Statement

All datasets generated for this study are included in the article/supplementary material.

## Ethics Statement

Written informed consent was obtained from the minor(s)' legal guardian/next of kin for the publication of any potentially identifiable images or data included in this article.

## Author Contributions

SC-C, NP-R, and PM-S coordinated all work and did most of the writing. JP-B was responsible for the evaluation of medical records and ultrasonographic data. HB-P was responsible for macroscopy pathology data. DP-E and NV-G were responsible for biochemical and genetics data. All authors reviewed and commented on drafts and approved the final manuscript and the decision to submit for publication.

## Conflict of Interest

The authors declare that the research was conducted in the absence of any commercial or financial relationships that could be construed as a potential conflict of interest.
